# Prevalence, distribution, and phylogenetics of the tick-borne relapsing fever spirochete *Borrelia turicatae* in the soft tick *Ornithodoros turicata americanus* in Florida

**DOI:** 10.1371/journal.pntd.0014473

**Published:** 2026-06-29

**Authors:** Nicholas Canino, Carson W. Torhorst, Sebastian Botero-Cañola, Kristen N. Wilson, Samantha M. Wisely

**Affiliations:** Department of Wildlife Ecology and Conservation, University of Florida, Gainesville, Florida, United States of America; Mahidol Univ, Fac Trop Med, THAILAND

## Abstract

In the southwestern United States, the occurrence and potential for disease spread of Tick-Borne Relapsing Fever (TBRF) has been studied. In Florida, *Borrelia* pathogens that cause TBRF have only been found in two domestic dogs (*Canis lupus familiaris*), yet the soft tick vector, *Ornithodoros turicata americanus*, is commonly found throughout the state. The goal of our study was to provide the first large-scale investigation of this disease system in the southeastern US. Our objectives were to: 1) describe the occurrence and prevalence of *Borrelia* spp. in ticks found throughout their distribution; and 2) phylogenetically describe the pathogen species compared to other isolates of *B. turicatae*. We pooled ticks by sample location and extracted DNA from over 3,000 ticks systematically collected throughout Florida. Conventional PCR was used with a genus-wide IGS primer to detect any *Borrelia* spp. present in the ticks. We discovered a low but detectable prevalence (7/745; 0.94%) of the pathogen within localized foci, which could present an epidemiological risk to humans and companion animals in those areas. We Sanger sequenced the 7 pools that were positive for *Borrelia* spp. and created a phylogenetic tree with our samples and 27 previously described isolates. Our tree showed clustering of our samples into two distinct clades, one that fit with Texas isolates and one that was entirely distinct. We hypothesized that a combination of biogeographic and host influences may be the driving force behind the history of *Borrelia turicatae* in Florida. Future research is needed to improve our understanding of the drivers of pathogen occurrence and the phylogeography of this species. By understanding the occurrence and phylogenetics of *Borrelia turicatae* in the state, we can better comprehend and mitigate the risk of this neglected vector-borne disease for humans and companion animals in Florida.

## Author summary

There is a large gap in knowledge regarding the bacteria that causes Tick-borne Relapsing Fever in the United States (US). This disease has significant public health concern as it can cause severe neurological symptoms, such as meningitis, encephalitis, and facial palsy. One of the etiological agents, *Borrelia turicatae*, has been reported throughout the southwestern United States and Central America; however, little is known of its presence in the southeastern US. In the 1990s, two dogs residing in north central Florida became infected with spirochaete bacteria that closely resembled *Borrelia*, and one dog was confirmed to have had *B. turicatae* a decade later. The soft ticks that transmit these bacteria are also found ubiquitously in Florida. Despite the widespread distribution of this vector in Florida, there has been no research conducted to determine the occurrence of the pathogen in soft ticks. Our research described the first records of this pathogen in Florida soft ticks and provided preliminary insight into how this pathogen became established in the state. Although ticks were collected from throughout the state, occurrence of *B. turicatae* was only from north central Florida. More research is needed to better understand the ecology of this pathogen and the risk it may pose to humans and companion animals.

## Introduction

Vector-borne zoonotic diseases are caused by a diverse group of pathogens that have become an increasingly relevant threat to public health globally. This vigilance is especially needed for vector-borne pathogens that can persist in the environment through sylvatic cycles and can re-emerge in human populations [1,2]. In the United States, the threat of vector-borne diseases has increased significantly as human populations continue to encroach on already fragmented and degraded ecosystems [3]. These conditions enhance human exposure to vectors and the pathogens they harbor, creating favorable environments for disease emergence.

Tick-borne borrelioses are increasingly prevalent vector-borne diseases that include the extensively studied Lyme Borreliosis, as well as Tick-borne Relapsing Fever (TBRF). Globally, TBRF is a neglected disease that often goes underreported in humans and whose environmental and biological reservoirs are understudied [4,5], yet TBRF is often considered an important cause of acute undifferentiated febrile illnesses (AUFI) throughout the temperate and tropic regions of the world [6].

Tick-borne Relapsing Fever is characterized by recurrent febrile episodes that last between 2–7 days with afebrile periods of up to 10 days [7]. An infected individual may experience as many as 12 episodes with complicating symptoms becoming increasingly likely after the second episode. These symptoms are generally unspecific such as headache, myalgia, nausea, and chills, but can also include severe neurological signs, such as meningitis, encephalitis, and facial palsy. *Borrelia turicatae* is one of the few *Borrelia* species reported to be neurotropic [8]. In general, RF-group *Borrelia* infections are readily treatable with proper antibiotic regimens, but the disease remains neglected and is frequently underdiagnosed or misdiagnosed in clinical settings [9].

The primary vector of TBRF-group *Borrelia* around the world are ticks in the family Argasidae (Acari). In the United States, these pathogens are transmitted by soft tick vectors in the genus *Ornithodoros*. These ticks are generalist feeders and have been reported feeding on all classes of terrestrial vertebrates [10]. Soft ticks are nidicolous, meaning they spend their lives in nests, burrows, and crevices, and are attached to their hosts for short periods of time [10]. Unlike their hard-bodied counterparts that feed on a host for long periods and are therefore often collected while attached to their vertebrate hosts, soft ticks can take a complete bloodmeal and detach from their host in under an hour. Thus, soft ticks are found less frequently on their hosts. As a result, these ticks are significantly understudied compared to hard ticks, and the threat of disease to humans from these vectors are not entirely understood, which contributes to their status as neglected vectors [11].

**Fig 1:**
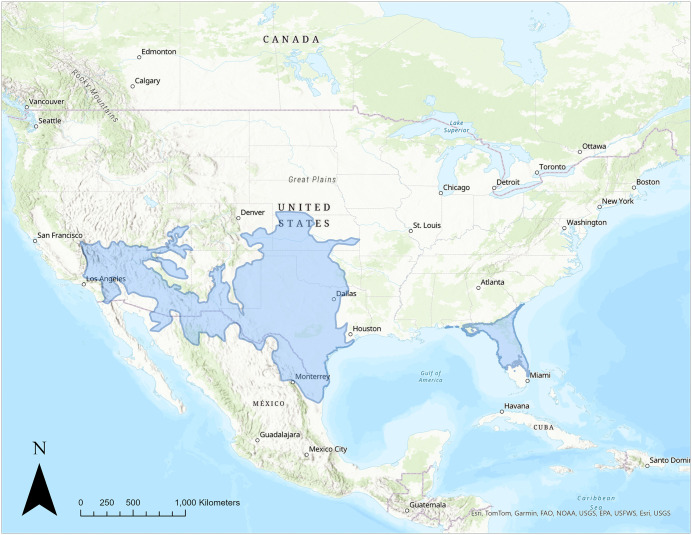
Distribution of the soft tick *Ornithodoros turicata* in North America. The range of the species in the western population was determined by Donaldson et al. [14] and in the eastern population by Botero-Cañola et al. [15]. Basemap: World Topographic Map (US Edition). Sources: Esri, TomTom, Garmin, FAO, NOAA, USGS, **(c)** OpenStreetMap contributors, and the GIS User Community. https://www.arcgis.com/home/item.html?id=668f436dc2dc4f2c83ceb0c064380590.

The soft tick species *Ornithodoros turicata* is a common species in the United States and has been documented feeding on numerous wildlife, domestic species, and humans [12,13]. The distribution of this species primarily includes central and western Texas and parts of Oklahoma and northern Mexico in the west, and most of Florida in the east [14,15] (Fig 1). These disjunct populations are separated on either side of the Mississippi River Valley [10]. In the southwestern US and Mexico, this tick occurs in a variety of microhabitats including caves from where most human cases of TBRF have been recorded. In Florida, the subspecies *O. turicata americanus* has only been found in gopher tortoise (*Gopherus polyphemus*) burrows and has not been reported feeding on humans nor have human cases of TBRF been reported in the state [16].

*Ornithodoros turicata americanus* is understudied compared to the western population of the species and an association of this tick subspecies with *Borrelia turicatae* has not been observed. The two disjunct subspecies of this soft tick have numerous ecological and biological differences which makes studying both populations important. Besides showing distinct microhabitat selection, the two tick subspecies also vary in their competence as vectors of different *Borrelia turicatae* strains. Laboratory research has shown that soft ticks from the southwestern US can transmit both the Florida and Texas strains of *B. turicatae* whereas the Florida soft ticks are a more competent vector for the Florida strain [16]. These differences highlight the need for further research on *O. turicata* to better understand the ecology of the subspecies and the impacts these differences may have on public and veterinary health.

In 1990 and 1992, two separate cases of spirochetemia were reported in domestic dogs in north central and central Florida [17]. The first case was in Alachua County, where the owner reported that the dog frequently roamed throughout upland pine habitat on the property. This habitat is one of the most suitable for gopher tortoises and likely has a high abundance of soft ticks [15,18]. There was no previous reports of travel and the dog was often found infested with ticks. The second dog resided in Sumter County and also frequently roamed a large property where it became persistently infested by ticks. The spirochete causing infection in this dog was successfully cultured and identified as a Florida Canine Borrelia (FCB). Future studies using this isolate were able to confirm that it was *B. turicatae*, which made it the first record of this pathogen in Florida [19]. Despite this, further research was not pursued to determine the origin of infection in the dogs nor the presence of the bacteria in soft ticks in the state.

*Ornithodoros turicata americanus* occurs almost exclusively in Florida and is distributed throughout most of the peninsula and panhandle. Both canine *Borrelia* cases occurred within the known distribution of the vector [15]. Although the occurrence of the putative vector and canine TBRF cases in Florida has been documented, there is a gap in the data that directly links *O. t. americanus* and *B. turicatae in situ*. We hypothesized that *B. turicatae* would be found in *O. t. americanus* at low but detectable levels in Florida. This research provides needed information on this neglected vector and pathogen system. The objectives of this study were to: 1) describe the occurrence and prevalence of TBRF *Borrelia* sp. in soft ticks collected throughout its distribution in the state; and 2) genetically characterize the TBRF *Borrelia* sp. in order to infer the relationship with *Borrelia* sp. across the country. This research provides a better understanding of disease risk from soft ticks and can inform risk mitigation strategies for this vector-borne disease for humans and companion animals in Florida.

## Materials and methods

### Field and laboratory collection

Because of the known association of *O. t. americanus* and gopher tortoise burrows, we surveyed burrows using a random design that was stratified by ecoregion. The resulting survey locations covered the distribution of the species and equally sampled the major ecoregions within the distribution. Surveys were confined to public land with known gopher tortoise populations. We surveyed 102 sites across the state plus 11 post hoc sites to improve geographic variation [15]. At each site we surveyed for soft ticks at 5 gopher tortoise burrows spaced at least 50 m apart. If a site only had burrows within 50 m from each other, we sampled those that were at the furthest distance from each other. Prior to conducting field work, permits were obtained from the Florida Fish and Wildlife Conservation Commission to sample around and within gopher tortoise burrows (Permit N. LSSC-22-00054). Because this work did not involve handling vertebrates or engaging in activities that altered the behavior of vertebrates, an Institutional Animal Care and Use (IACUC) protocol at the University of Florida was not required.

**Fig 2:**
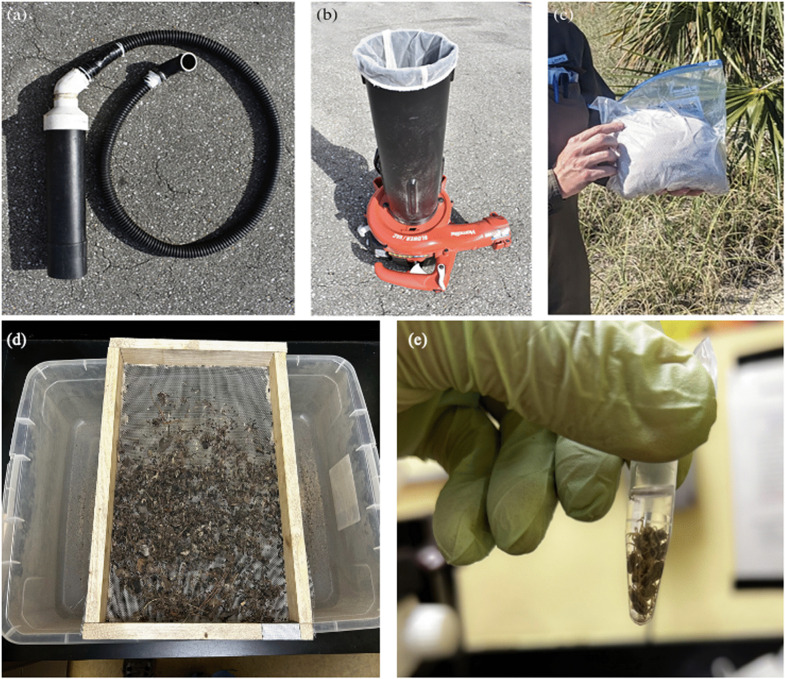
Depiction of standardized system used to collect soft ticks across Florida. **(a)** A 4-cm internal diameter hose attached to the lower vacuum tube through a PVC elbow and adapter. The posterior end of the hose included a rubber elbow to facilitate sample collection. **(b)** A Homelite 26 cc gas-powered blower/vacuum with collection mesh installed on the upper vacuum tube. **(c)** One 3.8-L resealable polyethylene bag containing the mesh with approximately 2000 g of the collected burrow material. **(d)** A 1.6 mm mesh screen with burrow material sifted through to a plastic bin. Burrow material was examined and soft ticks collected before material in sieve was disposed of and material in bin was passed through the next screen. **(e)** A 1.5 mL microcentrifuge tube with 95% molecular grade EtOH and soft ticks that were collected from a single burrow sample.

We developed a standardized protocol for rapid and reliable collection of soft ticks from gopher tortoise burrows (Fig 2) [20]. At each burrow, a borescope was used to assess the presence of vertebrate species within the first 2 m of the burrow. A burrow was not sampled if there was a vertebrate present. At empty burrows, we used a gas-powered vacuum to extract substrate from the first meter of the gopher tortoise burrow and then used sieves to separate ticks from substrate samples. Sampling occurred from October 2022 to May 2023 with one tick collected opportunistically from a burrow in November 2024 while repeating sampling efforts for collaborators.

To sample each burrow, we used the vacuum to fill the mesh bag with substrate material and then placed the mesh bag inside a 3.8-L Ziploc bag. Samples from each burrow were weighed and inspected for soft ticks at the seams and ensured an airtight seal before processing began. We immediately removed any soft ticks discovered and stored them in a 1.5 mL microcentrifuge (MC) tube containing 95% molecular-grade Ethanol (EtOH). To examine the collected substrate samples, a sieving system was developed that used three screens of decreasing mesh sizes (1.6, 1.2, and 1.0 mm). An ultraviolet (UV) hand-held light was used to detect the fluorescence in the legs of soft ticks [20]. We collected all soft ticks found during the sieving process and grouped ticks from the same burrow together in a single MC tube. All tubes were stored in a -20 ºC freezer. Any burrow material that remained was placed back in the sample bag, sealed, and discarded.

### Tick identification and DNA extraction

We characterized species, life stage, and sex (if adults) for 20% of the soft ticks that were collected using morphological keys [10]. We assumed the rest of the soft ticks to be *O. t. americanus* based on gross examination and their habitat specificity, as they are the only soft tick species to be recorded in gopher tortoise burrows [15]. We rinsed soft ticks of any large material debris using 95% EtOH and separated individuals into their own 1.5 mL MC tubes. DNA extraction was performed using a Gentra Puregene DNA Extraction Kit (Qiagen, Hilden, Germany) in a low yield/low molecular weight nucleic acid extraction room to reduce contamination. Ticks were first washed in a 10% bleach solution for 15 seconds followed by two washes in DI water for an additional 15 seconds each. This process removed environmental DNA and contaminants from the exterior before extraction. After washing, each tick was ventrally bisected using a flame sterilized blade. Ticks were pooled by burrow in groups of a maximum of 5 individuals and placed in a 1.5 mL MC tube containing 600 µL of Puregene Cell Lysis solution for at least 24 hours. Proteinase K (20 µL, Millipore Sigma, Darmstadt, Germany) was added to each vial and incubation in this solution was performed at 56 ºC for a minimum of 16 hours under gentle mixing. The remaining extraction process was completed using the manufacturer’s protocol. An aliquot of 20 µL of extracted DNA was kept in short-term storage in 4 ºC until analysis and the remaining extracted DNA was kept in long-term storage in -20 ºC.

### *Borrelia* screening and sequencing

We screened pools of soft ticks for the presence of *Borrelia* DNA using conventional polymerase chain reaction (PCR). We amplified an intergenic spacer gene located between the 16S (rrs) and 23S (rrIA) ribosomal RNA; this sequence is conserved across the genus (IGS; S1 Table). We used a nested PCR with the IGS primer and a Taq PCR Master Mix (Qiagen, Hilden, Germany) according to the protocols described in Bunikis et al. [21]. Other than the specified cycle conditions of the protocol, we included an initial denaturation step of 95 ºC for 2 minutes and ended with 10 minutes of elongation at 72 ºC. The initial denaturation and final elongation steps were also included in the nested reaction (S2 Table). Samples that underwent PCR were visualized using 2% agarose gel electrophoresis. Positive samples were gel extracted and purified using the Zymoclean Gel DNA Recovery Kit (Zymo Research, USA), and the products were sent to McLabs (San Francisco, CA, USA) for Sanger Sequencing.

### Statistical analysis

To determine statewide and local prevalence of *Borrelia*, we used a Bayesian framework to estimate the true prevalence using the truePrevPool function in the *prevalence* package in R [22]. This function used the apparent prevalence and the variance in size of the tick pools to model the true prevalence at the state and site level. There was no information in the literature that describes the test characteristics of the IGS primer; thus, we tested the model using a beta distribution centered around 80% for sensitivity (α=24.8, β=6.2) and a fixed 99% specificity level as suggested by the package.

We constructed a phylogenetic tree using 7 sequences for the IGS amplicon of *B. turicatae* derived from this study and 26 sequences of *B. turicatae* isolates originating in different hosts from Florida, Kansas, and Texas published on NCBI GenBank (S3 Table). We also obtained an IGS sequence of a *B. hermsii* isolate from NCBI GenBank to use as an outgroup. Sequences were aligned using the MUSCLE alignment algorithm and phylogenetic trees were assembled using the Mega12 software. We constructed a phylogenetic tree using a maximum likelihood method with a Tamura 3-parameter substitution model with invariant rates among sites. We generated estimates of our phylogeny using a standard bootstrap analysis with 1,000 replicates. Branches with supporting bootstrap values below 70% were collapsed using TreeCollapseCL4 to avoid interpretation of weakly-supported branching [23].

## Results

### Soft tick collection

We sampled 591 burrows across 113 sites and successfully collected soft ticks from 203 burrows (34%) at 61 of the sites (54%). At sites where soft ticks were detected, an average of 3 burrows were infested with ticks. The average abundance of soft ticks at each burrow was 15 ± 2.3 soft ticks (Range: 1–232). A total of 3,066 soft ticks were collected throughout the state and all 631 ticks that underwent morphological identification were *Ornithodoros turicata americanus*. A subset of these ticks were identified to life stage that included 19 larva (3%), 524 nymphs (83%), and 88 adults (14%).

### *Borrelia* screening

**Fig 3:**
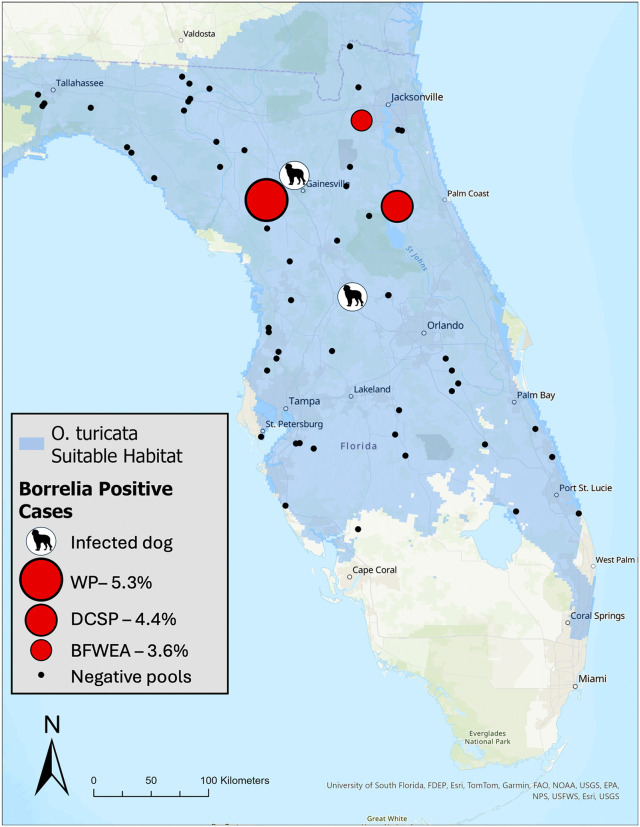
The suitable habitat of *Ornithodoros turicata americanus* as described in Botero-Cañola et al. (2024) with all the sites where soft ticks were collected. Black sample points were sites where soft ticks were collected but *Borrelia* was not identified. The positive sites were size scaled based on the prevalence of the pathogen at the site. The dog symbol represents the approximate location of the spirochetemic domestic dogs described in [17]. The statewide prevalence was 1.9%. Basemap: World Topographic Map (US Edition). Sources: Esri, TomTom, Garmin, FAO, NOAA, USGS, **(c)** OpenStreetMap contributors, and the GIS User Community. https://www.arcgis.com/home/item.html?id=668f436dc2dc4f2c83ceb0c064380590. Dog Silhouette by Margot Michaud, from PhyloPic (https://www.phylopic.org), licensed under CC0 1.0.

We pooled soft ticks into 745 pools with the mean number of ticks per pool being 4 ± 0.06 and over 500 pools containing the maximum 5 ticks. All 745 pools were screened for *Borrelia* sp. using the pan-*Borrelia* primer, IGS, and 7 pools (0.94%) collected from 3 sites amplified the *Borrelia* gene target. Four of the pools were collected from Dunns Creek State Park (DC) near Palatka, Florida, two pools were collected at Branan Field Wildlife and Environmental Area (BF) near Jacksonville, Florida, and the one pool was collected from Watermelon Pond Wildlife and Environmental Area (WP) in Newberry, Florida (Fig 3). All positive pools from each site originated from only one burrow. The true prevalence within site was highest at WP (5.3%; 95% CI: 1.3- 11.4%), then DC (4.4%; 95% CI: 2.3-7.2%), and lastly, BF (3.6%; 95% CI: 1.4-6.7%) had the lowest prevalence. The true statewide prevalence was 1.9% (95% CI: 1.2-2.9%).

**Fig 4:**
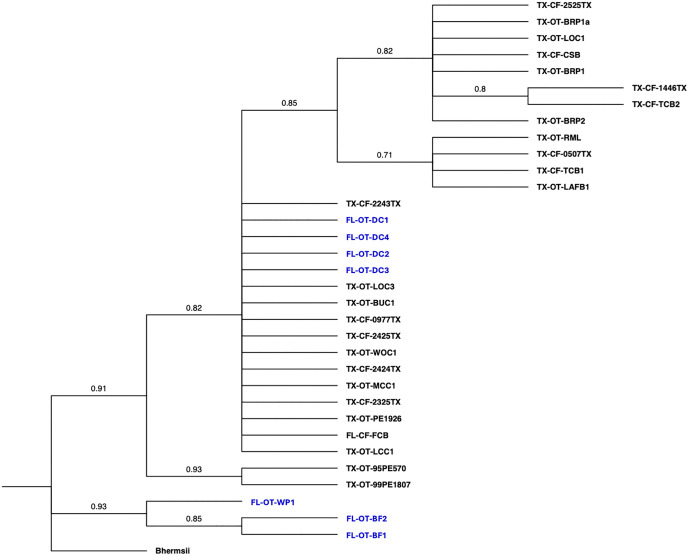
Phylogram of the Intergenic Spacer Gene (IGS) for isolates of *Borrelia turicatae* (black) and 7 samples collected from Florida in this study (blue, GenBank Accession No: PX741051- PX741057). This tree was developed using a maximum-likelihood model (bootstrap replicates = 1,000). States are abbreviated as: FL = Florida; TX = Texas; KS = Kansas. Originating hosts are abbreviated as: OT = *Ornithodoros turicata*; CF = *Canis familiaris*. Sample Sites are abbreviated as: DC = Dunns Creek State Park, WP = Watermelon Pond Wildlife and Environmental Area, BF = Branan Field Wildlife and Environmental Area.

The proximity in the phylogenetic tree of *B. turicatae* genetic sequences collected from dogs in previous studies and from ticks in the present study suggest that these samples were genetically similar (Fig 4). The tree had strong supporting values for delineation of the major clades; however, branching of individual isolates and samples was not supported by bootstrapping values. We therefore left the phylogenetic relationship between individuals within a clade unresolved. The phylogenetic tree showed five distinct clades, four of which correspond to similar findings in Krishnavajhala et al. [9]. A fifth clade consisted of new sequences described in this study which were distinct from previously described sequences. Samples from one site in Florida fit into the clade that also included the Florida Canine Borrelia and numerous isolates from Texas. Samples from the other two sites fit into a clade distinct from other previously described isolates. Thus, our samples of *Borrelia turicatae* are separated into two distinct clades, one of which was unique from other published isolates.

## Discussion

### Key findings and implications

This study described the first records of *Borrelia turicatae* in soft ticks in Florida. We demonstrated that there was a low but measurable prevalence of this pathogen distributed in localized foci in north central Florida. We did not confirm *B. turicatae* presence in soft ticks in other parts of its range. The only previous record of Tick-borne Relapsing Fever in Florida was in domestic dogs in the 1990s [17]. Both of these dogs lived in habitat that was suitable for both gopher tortoises and the soft ticks that inhabit their burrows. In this study, we detected *B. turicatae* in ticks residing in burrows near these canine cases. Although exact locations of the dogs were not publicly available, the Alachua County dog was presented to a veterinary clinic within 30 km of the Watermelon Pond site where infected soft ticks were confirmed. Interestingly, we did not find any evidence of soft ticks harboring *B. turicatae* within 100 km of the Sumter County dog case. Since this dog’s history demonstrated constant tick infestation in suitable habitat, this may suggest that soft ticks are carrying the pathogen in areas south of the foci described in this study.

The Florida strains of *Borrelia* fit into two distinct clades within *B. turicatae*. The Branan Field and Watermelon Pond samples were genetically distinct from other described sequences of *B. turicatae*, but the Dunns Creek samples fit into the same clade as the Florida Canine Borrelia and numerous Texas isolates. This pattern provides evidence that Florida soft ticks are potentially carrying two distinct strains of *B. turicatae*, and the pattern may be the result of two phenomenon: a historical isolation event that drove the divergence of these strains and an admixture event that brought Texas strains into Florida in more recent years.

We hypothesize that the BF and WP samples of *Borrelia* represent a divergent strain of the bacteria that resulted from the disjunct and isolated distribution of the two soft tick subspecies over geologic time. There is strong evidence that the two subspecies of *O. turicata* arose due to isolation by the Mississippi River Delta [10,15]. This river delta formed as long as 80 million years ago [24]. Biogeographic separation caused by the Mississippi River delta has been documented as a driver of divergence for numerous mammal and fish species [25,26]. Although no research has documented this in an invertebrate vector, it is likely that the same phenomenon drove differentiation in *O. turicata* and its associated microorganisms. This isolation could have led to a Florida strain of *B. turicatae* developing within the isolated Florida soft tick subspecies.

The Dunns Creek samples and Florida Canine Borrelia isolate represent strains of *B. turicatae* that fall within the same clade as western isolates. This may be a result of an admixture event caused by the introduction of western strains back into Florida in the recent past. *O.* *turicata* feed on a wide variety of migratory and mobile species. Vertebrate hosts of *B. turicatae* may disperse the pathogen far beyond local foci and new strains may emerge within geographically distant vector populations. Coyotes (*Canis latrans*) represent a strong candidate for a vertebrate host that may drive the spread of *Borrelia turicatae* strains from Texas into Florida. This canid species has been implicated as a reservoir host of *B. turicatae* and are known for their large home ranges and expanding eastward distribution [27]. The eastward expansion of coyotes into Florida from the southwestern United States beginning in the 1960s may have introduced this pathogen strain into soft ticks [28]. The introduction of western strains may have resulted in a recent admixture between the two populations, which is strongly supported by the FCB and DC strains clustering in the same clade as Texas isolates.

Phylogenetic analysis indicated that the bacteria found in ticks was similar to the Florida Canine Borrelia that was isolated from one of the dogs. These dogs both presented with serious clinical signs consistent with spirochetemia, including anorexia, leg lameness, stiff gait, and anemia. At least one dog did not receive treatment until 7 weeks after initial visit because of the lack of knowledge surrounding TBRF Borrelia in Florida. Research in Texas shows that *Borrelia* infection in dogs may be a larger issue than previously understood and the same case may be true in Florida as well [29]. Thus, more research is needed to understand the potential health threats that domestic dogs with access to these habitats face and more awareness needs to be brought to pet owners and veterinarians.

### Limitations

Our study had several limitations. Insufficient sampling may have biased surveillance for *B. turicatae*. Sites with detection of *Borrelia* were among the areas with the greatest number of ticks collected (DC = 1st, BF = 3rd, and WP = 10th). Thus, it is likely that our ability to detect pathogen presence at a site was related to the quantity of ticks collected from that site. However, it has been shown that pathogen prevalence is associated with vector abundance, and as a result, we cannot determine whether reported prevalence levels were influenced by the probability of detection or were the result of natural vector-pathogen dynamics [30]. Interestingly, most of the other sites with high abundance of soft ticks were also located in north central Florida but did not have any *Borrelia* detections. More research is needed to understand the occurrence of this pathogen in this part of the state and the mechanisms that drive pathogen foci to develop in certain locations.

The phylogenetic analysis in our study was limited by the use of only one gene locus. Less phylogenetic information can be extrapolated from a single gene as opposed to multiple locus or a whole genome, and as a result our phylogenetic tree did not have strong support for resolving evolutionary relationships beyond major clades. Multiple loci will be needed to resolve the relationship within clades and better understand the lineages of this bacterial species. Despite using only one gene, the tree strongly supported clustering of our samples into two distinct clades and permitted us to develop phylogeographic hypotheses describing the pattern of divergence of two *B. turicatae* strains. The development of isolates originating from Florida soft ticks in the future may provide the opportunity to compare differences in pathogenicity and infectivity between western and Florida strains.

### Public health relevance

Tick-borne Relapsing Fever is a neglected disease around the world, but especially in the United States where it is overshadowed by Lyme Disease. TBRF is reportable in twelve states, yet it is underdiagnosed because of lack of commercially available tests and awareness of the disease [31]. Currently, TBRF is not a reportable disease in Florida. Since *O. turicata americanus* are nidicolous and live solely in gopher tortoise burrows, it is possible that the risk of exposure to humans is low. Nonetheless, there are certain groups that may have elevated risk of exposure due to their proximity and/or behaviors around gopher tortoise burrows. Rural communities throughout Florida occur in gopher tortoise habitat [32]. Private landowners are pivotal for gopher tortoise conservation in the southeast and many landowners have tortoise burrows on their property [32]. These landowners would benefit from educational campaigns that impress the importance of not approaching or manipulating gopher tortoise burrows which helps conserve the species and protects human health. Wildlife biologists and technicians who conduct research or management activities near or within gopher tortoise burrows also need to be aware of the vectors and health threats associated with gopher tortoise burrows. A review of two major standardized operating procedures for surveying gopher tortoise burrows did not mention the use of personal protective equipment nor the risk of disease transmission associated with gopher tortoise burrows [33,34]. Additionally, conversations with gopher tortoise researchers confirm the general unfamiliarity with disease risk from these burrows (per comms.). Fortunately, most researchers were open to learning more about Tick-borne Relapsing Fever and adapting their practices to protect themselves from this disease. Vector-borne diseases are becoming an increasingly important threat worldwide, and an openness to mitigation strategies can improve public health outcomes.

As human populations continue to encroach on wildlands, we need to improve our understanding of the disease systems and their vectors that exist in these habitats. Soft ticks are an understudied group of vectors that can exist close to human settlements and carry pathogens that cause notable disease in humans. These ticks are difficult to detect which may be why Tick-borne Relapsing Fever continues to be a neglected disease. TBRF can cause severe febrile episodes and neurological signs in humans and misdiagnosis likely occurs nationwide, and therefore improving research and awareness efforts is critical. Our study provides the baseline for Florida’s soft tick-*Borrelia* ecology research by uncovering potential patterns in prevalence and describing aspects of this pathogen’s phylogenetic history. Future work needs to focus on filling the knowledge gaps regarding endemicity, transmission dynamics, and the factors that increase spillover risk into domestic dogs and humans.

## Supporting information

S1 Table Oligonucleotide sequences developed by Bunikis et al. [21] and used as primers for a nested conventional PCR in this study. (DOCX)

S2 Table Thermocycler conditions used for the nested PCR reaction developed by Bunikis et al. [21]. Our study included an initial denaturation and final extension step as recommended by the producers of the *Taq* PCR Master Mix (Qiagen, Hilden, Germany).
(DOCX)

S3 Table IGS sequences obtained from GenBank of *B. turicatae* isolates from Texas, Florida, and Kansas. IGS sequences obtained from our samples are in blue. One *B. hermsii* isolate was obtained for use as an outgroup.
(DOCX)
