# Supplementary material for: Prevalence, distribution, and phylogenetics of the tick-borne relapsing fever spirochete Borrelia turicatae in the soft tick Ornithodoros turicata americanus in Florida
Source: PLoS Negl Trop Dis. 2026 Jun 29;20(6):e0014473. doi: 10.1371/journal.pntd.0014473 (PMC13327513; doi:10.1371/journal.pntd.0014473)
Supplement: S1 Table — (DOCX) [file pntd.0014473.s001.docx]

**S1 Table**. **Oligonucleotide sequences developed by Bunikis et al. [22] and used as primers for a nested conventional PCR in this study.**

| Primer^a^ | Gene Locus | Sequence (5’-3’) |
| --- | --- | --- |
| **16S-23S IGS^b^** | ***rrs-rrIA Intergenic Spacer*** |  |
| IGS F1 |  | GTATGTTTAGTGAGGGGGGTG |
| IGS R1 |  | GGATCATAGCTCAGGTGGTTAG |
| IGS Fn |  | AGGGGGGTGAAGTCGTAACAAG |
| IGS Rn |  | GTCTGATAAACCTGAGGTCGGA |
| a. F1, first forward; R1, first reverse; Fn, nested forward; Rn, nested reverse.  b. Bunikis et al., 2004 | | |
