# Supplementary material for: Prevalence, distribution, and phylogenetics of the tick-borne relapsing fever spirochete Borrelia turicatae in the soft tick Ornithodoros turicata americanus in Florida
Source: PLoS Negl Trop Dis. 2026 Jun 29;20(6):e0014473. doi: 10.1371/journal.pntd.0014473 (PMC13327513; doi:10.1371/journal.pntd.0014473)
Supplement: S2 Table — Our study included an initial denaturation and final extension step as recommended by the producers of the Taq PCR Master Mix (Qiagen, Hilden, Germany). (DOCX) [file pntd.0014473.s002.docx]

**S2 Table. Thermocycler conditions used for the nested PCR reaction developed by Bunikis et al. [22]. Our study included an initial denaturation and final extension step as recommended by the producers of the *Taq* PCR Master Mix (Qiagen, Hilden, Germany).**

| **Cycle Step** | **Temperature (ºC)** | | **Time** | **Number of Cycles** |
| --- | --- | --- | --- | --- |
|  |  |  | |  |
| *Inside Reaction* |  |  | |  |
| Initial Denaturation | 95 | 2 min | | 1 |
| Denaturation  Annealing  Extension | 94  56  74 | 30 s  30 s  60 s | | 35 |
| Final Extension | 72 | 10 min | | 1 |
| Hold | 10 | ∞ | | 1 |
|  |  |  | |  |
| *Outside Reaction* |  |  | |  |
| Initial Denaturation | 95 | 2 min | | 1 |
| Denaturation  Annealing  Extension | 94  60  74 | 30 s  30 s  60 s | | 40 |
| Final Extension | 72 | 10 min | | 1 |
| Hold | 10 | ∞ | | 1 |
