# Supplementary material for: Prevalence, distribution, and phylogenetics of the tick-borne relapsing fever spirochete Borrelia turicatae in the soft tick Ornithodoros turicata americanus in Florida
Source: PLoS Negl Trop Dis. 2026 Jun 29;20(6):e0014473. doi: 10.1371/journal.pntd.0014473 (PMC13327513; doi:10.1371/journal.pntd.0014473)
Supplement: S3 Table — IGS sequences obtained from our samples are in blue. One B. hermsii isolate was obtained for use as an outgroup. (DOCX) [file pntd.0014473.s003.docx]

**S3 Table. IGS sequences obtained from GenBank of *B. turicatae* isolates from Texas, Florida, and Kansas. IGS sequences obtained from our samples are in blue. One *B. hermsii* isolate was obtained for use as an outgroup.**

| **Sequence ID^a^** | **Accession** | **Host source** | **Locality^b^** | **Reference** |
| --- | --- | --- | --- | --- |
|  |  |  |  |  |
| *B. turicatae* |  |  |  |  |
| FL-CF-FCB | DQ855552.1 | Domestic dog | Sumter Co., FL | Schwan et al., 2004 |
| TX-OT-LCC1 | MT682898.1 | Soft tick | San Antonio, TX | Krishnavajhala et al., 2021 |
| TX-OT-MCC1 | MT682899.1 | Soft tick | San Antonio, TX | Krishnavajhala et al., 2021 |
| TX-OT-WOC1 | MT682900.1 | Soft tick | San Antonio, TX | Krishnavajhala et al., 2021 |
| TX-OT-LAFB1 | MT682908.1 | Soft tick | San Antonio, TX | Krishnavajhala et al., 2021 |
| TX-OT-LOC1 | MT682901.1 | Soft tick | Austin, TX | Krishnavajhala et al., 2021 |
| TX-OT-LOC3 | MT682902.1 | Soft tick | Austin, TX | Krishnavajhala et al., 2021 |
| TX-OT-BUC1 | MT682903.1 | Soft tick | Austin, TX | Krishnavajhala et al., 2021 |
| TX-CF-CSB | MT682909 | Domestic dog | College Station, TX | Krishnavajhala et al., 2021 |
| TX-OT-BRP1 | MT682904.1 | Soft tick | Austin, TX | Bisset et al., 2018 |
| TX-OT-BRP1a | MT682905.1 | Soft tick | Austin, TX | Bisset et al., 2018 |
| TX-OT-BRP2 | MT682906.1 | Soft tick | Austin, TX | Bisset et al., 2018 |
| TX-CF-TCB1 | DQ855554.1 | Domestic dog | Clay Co., TX | Schwan et al., 2005 |
| TX-CF-TCB2 | DQ855558.1 | Domestic dog | Lubbock Co., TX | Schwan et al., 2005 |
| TX-OT-95PE570 | DQ855553.1 | Soft tick | Atascosa Co., TX | Schwan et al., 2005 |
| TX-OT-99PE1807 | DQ855556.1 | Soft tick | Real Co., TX | Schwan et al., 2005 |
| TX-OT-PE1926 | DQ855555.1 | Soft tick | Real Co., TX | Schwan et al., 2005 |
| KS-OT-RML | DQ855545.1 | Soft tick | Kansas, USA | Bunikis et al., 2004 |
| TX-CF-2325TX | MH620360 | Domestic dog | Randall Co., TX | Modarelli et al., 2019 |
| TX-CF-0507TX | MH620361 | Domestic dog | Coryell Co., TX | Modarelli et al., 2019 |
| TX-CF-1446TX | MH620362 | Domestic dog | Gillespie Co., TX | Modarelli et al., 2019 |
| TX-CF-0977TX | MH620363 | Domestic dog | Coryell Co., TX | Modarelli et al., 2019 |
| TX-CF-2243TX | MH620364 | Domestic dog | Amstrong Co., TX | Modarelli et al., 2019 |
| TX-CF-2424TX | MH620365 | Domestic dog | DeWitt Co., TX | Modarelli et al., 2019 |
| TX-CF-2425TX | MH620366 | Domestic dog | Caldwell Co., TX | Modarelli et al., 2019 |
| TX-CF-2525TX | MH620367 | Domestic dog | Gonzales Co., TX | Modarelli et al., 2019 |
| FL-OT-DC2 | PX741051 | Soft tick | Putnam Co., FL | current research |
| FL-OT-DC1 | PX741052 | Soft tick | Putnam Co., FL | current research |
| FL-OT-DC3 | PX741053 | Soft tick | Putnam Co., FL | current research |
| FL-OT-DC4 | PX741054 | Soft tick | Putnam Co., FL | current research |
| FL-OT-BF2 | PX741055 | Soft tick | Duval Co., FL | current research |
| FL-OT-BF1 | PX741056 | Soft tick | Duval Co., FL | current research |
| FL-OT-WP1 | PX741057 | Soft tick | Alachua Co., FL | current research |
| *B. hermsii* |  |  |  |  |
| Bhermsii-DAH | CP073136.1 | Human | Washington, USA | Kneubehl & Lopez, 2021 |
|  |  |  |  |  |
| a. FL, Florida; KS, Kansas; TX, Texas; CF, *Canis familiaris*; OT, *Ornithodoros turicata*.  b. Co., County. | | | | |
|  | | | | |
